# Chronic Disease Management in Sickle Cell Trait Patients in the Primary Care Setting: A Case Report

**DOI:** 10.7759/cureus.11255

**Published:** 2020-10-30

**Authors:** Rachel N Fields, Suzanne Minor

**Affiliations:** 1 Family Medicine, Florida International University Herbert Wertheim College of Medicine, Miami, USA; 2 Family Medicine, Office of Academic Affairs, Florida International University, Miami, USA

**Keywords:** sickle cell trait, fructosamine, preventive medicine, co-morbid conditions, chronic disease management, glycated hemoglobin a

## Abstract

Sickle cell disease (SCD) is a heterogeneous group of inherited hemoglobinopathies associated with mutations in the beta subunit of the hemoglobin protein. Several case reports and scientific reviews of the current literature have been published that indicate individuals having a single copy of the mutant sickle cell allele, known as sickle cell trait (SCT), can experience the same functional asplenia and increased risk of cerebrovascular accidents, kidney disease, cardiovascular effects, and veno-occlusive diseases as SCD patients when they are exposed to extreme conditions and stressful environments such as high-altitude, deep-sea diving, and intense physical activity. SCT also impacts the management of chronic illnesses such as diabetes mellitus. Here, we report a patient presenting for primary care follow-up after an SCT-related splenic infarction in order to emphasize the unique impact of SCT on long-term care and preventive medicine in the primary care setting.

## Introduction

Sickle cell disease (SCD) is a heterogeneous group of inherited hemoglobinopathies associated with mutations in the beta subunit of the hemoglobin protein. It can present with a variety of complications that revolve around the general pathophysiology of vaso-occlusive pain crises. As it was first described as an autosomal recessive condition, it was initially hypothesized that disease states and complications were only seen in patients homozygous for the mutant allele or for those determined to be compound heterozygotes with mutant alleles associated with other hemoglobinopathies (hemoglobin C disease, hemoglobin S/beta-thalassemia, etc.) [1-2]. Since the 1970s, many case reports have been published that indicate having a single copy of the mutant sickle cell allele, known as sickle cell trait (SCT), may not be a benign state as originally hypothesized [2-4]. New theories are emerging based on developing scientific data that SCD may, in fact, be a co-dominant condition and expression of just one of the hemoglobin (Hb) S alleles (Hb S is the hemoglobin produced as a result of the mutant allele) can result in complications. Multiple reports have identified individuals with SCT that have experienced vaso-occlusive crises, especially when exposed to extreme circumstances or conditions [5]. Elite-level athletes and scuba divers with SCT or SCT individuals that travel to or live in high-altitude locations have been reported to experience similar complications to homozygous SCD patients [3-5]. Case-control studies have shown that individuals with SCT carry a similar risk of venous thromboembolism and pulmonary embolism as their SCD counterparts [2,6]. Multiple case studies further support this by exemplifying cases of retinal vascular occlusion or splanchnic venous thrombosis in SCT patients [6-7]. As of 2015, at least five cases of splenic infarction in SCT patients, with no predisposing factors or extreme environments, have been reported, indicating that SCT patients may not have to be in high-stress situations to undergo an SCT-related complication [8].

SCT is the most common hemoglobinopathy in the United States, affecting one in 12 (8%-10%) of African American individuals [1,5]. Given the large number of SCT patients in the United States, physicians need to be prepared to handle acute complications and the long-term management of SCT-related complications. The management of comorbid conditions is also affected by the SCT genotype, as well as preventive medicine management, as these patients can experience the same functional asplenia and increased risk of cerebrovascular accidents, kidney disease, and cardiovascular effects as SCD patients [9].

## Case presentation

A 63-year-old Hispanic male, with a past medical history of type 2 diabetes mellitus (T2DM), stage I nonalcoholic fatty liver disease, hyperlipidemia, and erectile dysfunction, presented to the primary care clinic for follow-up after hospital admission for splenic infarction. The patient has no previous history of trauma to the area, recent infections, or prior abdominal surgeries. The patient began to have left upper quadrant (LUQ) pain while on a trip to Peru (at a high altitude) associated with shortness of breath and O2 saturation of 63%. The patient had never traveled to high altitudes before and had no recollection of any similar past events of shortness of breath. He was admitted to a local hospital and an abdominal ultrasound was performed, showing no remarkable findings that could explain LUQ pain and desaturation. The pain continued after he returned to the United States at sea level, causing him to go to the emergency room where computed tomography (CT) showed a 2 cm subcapsular splenic hematoma, grade 2. He underwent splenic artery embolization on hospital day 3 and was cleared for discharge on hospital day 5. The patient underwent evaluation for the etiology of the desaturation-related splenic hemorrhage while admitted. Echocardiogram, pulmonary function tests, and the four-extremity ultrasound were all within normal limits. Hematology was consulted to rule out infectious causes and malignant causes of splenic infarction. Blood was drawn for hemoglobin electrophoresis, and the patient was given a new diagnosis of SCT based on a hemoglobin S percentage of 42.6%. After an otherwise benign workup, the patient was diagnosed with splenic infarction secondary to splenic syndrome. While hospitalized, lab data indicated uncontrolled T2DM with glycated hemoglobin (HbA1c) of 8.2 mmol/mol. The patient was discharged with instructions to follow-up with gastroenterology for a repeat abdominal CT with contrast in four weeks and with his primary care physician (PCP) for diabetes management. His most recent HbA1c level recorded at his PCP office two months prior to hospitalization was also 8.2 mmol/mol. Prior to hospitalization, the patient was taking metformin 1000 mg PO twice daily, atorvastatin 40 mg po daily, and glipizide 5 mg PO daily. No other prescribed or over-the-counter medicines were reported by the patient. While hospitalized, 10 units of long-acting insulin glargine were added to his treatment regimen at bedtime. Full medicine reconciliation while admitted did not indicate the ingestion of any medications that could have contributed to the development of the splenic infarction.

## Discussion

In this case, the patient had no known personal or familial history of SCT or SCD, thus the evaluation for the etiology of the splenic rupture was initially negative until SCD was considered. The Journal of Genetic Counseling stated in 2018 that only 16% of individuals of childbearing age know their SCT status, indicating that a large percentage of the SCT patient population is unaware of their diagnosis and its impact on their health and the health of their offspring [10]. Although all states in the United States now follow newborn screening standards that include screening for SCT and SCD, as of 2018, only 37% of parents report receiving notice of the sickle-cell-trait status of their child [10]. From a preventive health standpoint, focusing on the new diagnosis of SCT, the patient needs to be treated for functional asplenia. Precautions need to be taken to prevent overwhelming post-splenectomy infections (OPSI). Although research indicates that the effect of SCT on the spleen is less than SCD, SCT is still associated with hyposplenism and current recommendations indicate vaccination against encapsulated bacteria: the p23-valent pneumococcal polysaccharide vaccination, H influenzae type B conjugate vaccine, and the meningococcal vaccine [11]. This patient received all of these vaccines during his hospital admission. Antibiotic prophylaxis has not been shown to be effective in the prevention of OPSI and the only other measure needed to be taken is patient education to contact his PCP if he develops an acute febrile illness [11].

The management of chronic illnesses and preventive health measures can be affected by an SCT diagnosis, and the patient may require closer monitoring by the primary care physician. Research indicates that SCT can worsen vascular dysfunction seen in patients with hyperlipidemia and T2DM [9]. The formation of advanced glycation end products promotes inflammation and reactive oxygen species formation while hyperlipidemia promotes further endothelial dysfunction [9]. These pathologic processes, along with the decreased nitrous oxide bioavailability and vascular dysfunction associated with insulin resistance, result in an impaired vascular reactivity. This vascular dysfunction is worsened by the SCT-related generation of reactive oxygen species, resulting in an increased risk for accelerated cardiovascular and renal pathologies related to T2DM and hyperlipidemia [9]. Tight control and monitoring of this patient’s comorbid conditions should be a priority.

HbA1c concentration is the most commonly utilized method for measuring and monitoring blood glucose concentrations and diabetes management. Several studies, including a retrospective cohort study published in The Journal of the American Medical Association (JAMA) in 2017, provide evidence that HbA1c concentrations are an inadequate technique for monitoring blood glucose management in SCT and SCD patients with T2DM [12-13]. In this study, when comparing two-hour oral glucose tolerance tests in SCT patients to a control non-SCT cohort, the HbA1c concentrations of the SCT patients were significantly lower than the HbA1c concentration of control patients who received the same values on the two-hour oral glucose tolerance test [13]. The cause of this inaccuracy of HbA1c measurements in SCT patients is still being studied, as research is conflicting, but two main hypotheses have been developed: either the shorter half-life of HbS compared to HbA (wild-type hemoglobin making up the majority of hemoglobin in healthy adults) limits glycation and increases red blood cell turnover or the presence of HbS causes assay interference in the current HbA1c measurement techniques [13]. Underestimation of HbA1c could result in a delay in the diagnosis and treatment of pre-diabetes and diabetes and, therefore, increased end-organ damage related to this pathology in SCT patients. Fructosamine, a measurement of total glycated serum protein, is another way to monitor blood glucose and diabetes management. Because it is not specific to red blood cells, data indicates it may be a more accurate measurement technique in SCD and SCT patients [12-14]. Glycated albumin and 1,5-anhydroglucitol levels are also options shown to be unaffected by HbS. However, these alternatives all carry their own limitations, and guidelines for blood glucose monitoring for SCT patients are still under development [14]. After hospitalization, this patient's blood glucose monitoring was transitioned from HbA1c to fructosamine. His first recorded fructosamine level was 256 umol/L (reference range 205-285) three months after discharge, indicating appropriate diabetes management. Of course, without previous fructosamine levels, there is an inability to compare this value after insulin was added during hospital admission to values prior to beginning insulin therapy.

Multiple hepatobiliary pathologies are associated with SCD, but no data indicate a correlation between SCT/SCD and fatty liver [15]. This patient has a diagnosis of fatty liver disease confirmed by right upper quadrant ultrasound. Management in this patient for the fatty liver does not change based on his SCT genotype. He is, however, at higher risk for hepatic pathologies due to his fatty liver diagnosis and should be encouraged to pursue lifestyle modifications, such as weight loss and abstaining from alcohol use for fatty liver management; he should also receive hepatitis A and hepatitis B vaccinations [15].

SCT patients require close monitoring of renal function. The partial oxygen pressure of the renal medullary environment is below the threshold for sickling and can be associated with infarction and hematuria related to papillary necrosis [16]. African-Americans with SCT have two times the prevalence of end-stage renal disease (ESRD) compared to individuals without the trait. Renal medullary carcinoma is a complication documented almost exclusively in SCT and SCD patients [16]. First described in 1995, renal medullary carcinoma occurs in the epithelium in kidney locations in which sickling is most pronounced. Due to the aggressive nature of renal medullary carcinoma, it is important that physicians providing care to SCT and SCD patients be aware of this condition and its presenting symptoms (abdominal/flank pain, gross hematuria) in order to initiate a prompt evaluation. There are currently no screening guidelines due to the rarity of the complication and lack of available patients for the study, but it is recommended that SCT or SCD patients with hematuria or flank pain be encouraged to seek medical attention at the first sight of these symptoms [16].

Meticulous eye examinations should be performed due to the increased risk of retinal changes and retinal artery/arteriole occlusion. Optometry examination guidelines have not yet been released. These patients may present on physical examination with vitreous hemorrhages, chorioretinitis, tortuous/dilated retinal veins, and retinal microaneurysms. Optometric exams should include a measurement of intraocular pressure and visual acuity as well as the evaluation of the anterior and posterior structures by fluorescein angiography [7].

SCT patients also require additional lifestyle education to lower the risk of having a vaso-occlusive crisis. The National Athletic Trainers Association released a statement in 2002 promoting increased screening and offering measures to decrease the risk of SCT-related exertional collapse. SCT patients are encouraged to participate in year-round activities that allow for a constant, steady-state of exertion and should not participate in activities or performance tests such as mile run, serial sprints, or other activities that could cause a sudden increase in exertional status. SCT patients are also encouraged to limit exertion while ill, dehydrated, or experiencing extreme heat or high altitude. These recommendations were supported by a similar statement from the American Heart Association and American College of Cardiology in 2015 [3,17]. Multiple case reports have identified SCT crises during basic training and other military activities in military recruits and personnel [18]. However, it is important to still encourage moderate physical fitness in these patients, as blood viscosity and markers of oxidative stress have been found to be lower in trained vs. non-trained SCT patients [18].

## Conclusions

This case contributes supporting data that SCT patients are at risk for vaso-occlusive crises in high physiologic stress environments and emphasizes the importance of considering SCT pathophysiology in the management of chronic illnesses in the primary care setting. Tight control of hyperlipidemia, coronary artery disease, hypertension, and T2DM is required to reduce the risk of SCT-related complications. Further studies and the development of T2DM screening and management guidelines are needed in order to optimize T2DM care in SCT patients. Preventive care and education must also be performed diligently in these patients, as they are at an increased risk for infections and adverse events in high-stress environments. Patients require close follow-up of vaccination status as well as periodic optometry visits. The development of guidelines needs to be addressed for some of the SCT/SCD-related complications such as retinal and renal pathologies. Extensive education on exercise safety and concerning symptoms of SCT-related complications and diseases is essential. A public health concern is also brought to light, as many patients with sickle cell-related disorders are living unaware of their diagnosis, placing them and their future generations at risk for SCT or SCD-related complications.
